# The effects of respiratory muscle training on respiratory function and functional capacity in patients with early stroke: a meta-analysis

**DOI:** 10.1186/s11556-024-00338-7

**Published:** 2024-02-22

**Authors:** Yun-Shan Zhang, Kai Zhang, Lang Huang, Jing-Xue Wei, Zi-Ting Bi, Jing-Hua Xiao, Jian Huang, Chao-Song Luo, Ying-Dong Li, Jia-Mei Zhang

**Affiliations:** 1 Department of Rehabilitation Medicine, The First Affiliated Hospital of Guangxi Medical University, Nanning, 530021 China; 2 Department of Rehabilitation Medicine, Sir Run Run Shaw Hospital of Zhejiang University, Hangzhou, 310016 China; 3 Cardiopulmonary Rehabilitation Center, Jiangbin Hospital of Guangxi Zhuang Autonomous Region, Nanning, 530000 China; 4 Department of Rehabilitation Medicine, The Guangxi Zhuang Autonomous Region Workers’ Hospital, Nanning, 530000 China; 5 Department of Rehabilitation Medicine, Guangxi International Zhuang Medicine Hospital, NanningNanning, 530000 China

**Keywords:** Early stroke, Respiratory muscle training, Respiratory function, Functional capacity

## Abstract

**Background:**

Respiratory muscle training is a continuous and standardized training of respiratory muscles, but the evidence of the effects on early stroke patients is not clear. This meta-analysis aimed to investigate the effects of respiratory muscle training on respiratory function and functional capacity in patients with early stroke.

**Methods:**

PubMed, Embase, PEDro, ScienceDirect, AMED, CINAHL, and China National Knowledge Infrastructure databases were searched from inception to December 8, 2023 for articles about studies that 1) stroke patients with age ≥ 18 years old. Early stroke < 3 months at the time of diagnosis, 2) respiratory muscle training, including inspiratory and expiratory muscle training, 3) the following measurements are the outcomes: respiratory muscle strength, respiratory muscle endurance, pulmonary function testing, dyspnea fatigue score, and functional capacity, 4) randomized controlled trials. Studies that met the inclusion criteria were extracted data and appraised the methodological quality and risk of bias using the Physiotherapy Evidence Database scale and the Cochrane Risk of Bias tool by two independent reviewers. RevMan 5.4 with a random effect model was used for data synthesis and analysis. Mean differences (MD) or standard mean differences (SMD), and 95% confidence interval were calculated (95%CI).

**Results:**

Nine studies met inclusion criteria, recruiting 526 participants (mean age 61.6 years). Respiratory muscle training produced a statistically significant effect on improving maximal inspiratory pressure (MD = 10.93, 95%CI: 8.51–13.36), maximal expiratory pressure (MD = 9.01, 95%CI: 5.34–12.69), forced vital capacity (MD = 0.82, 95%CI: 0.54–1.10), peak expiratory flow (MD = 1.28, 95%CI: 0.94–1.63), forced expiratory volume in 1 s (MD = 1.36, 95%CI: 1.13–1.59), functional capacity (SMD = 0.51, 95%CI: 0.05–0.98) in patients with early stroke. Subgroup analysis showed that inspiratory muscle training combined with expiratory muscle training was beneficial to the recovery of maximal inspiratory pressure (MD = 9.78, 95%CI: 5.96–13.60), maximal expiratory pressure (MD = 11.62, 95%CI: 3.80–19.43), forced vital capacity (MD = 0.87, 95%CI: 0.47–1.27), peak expiratory flow (MD = 1.51, 95%CI: 1.22–1.80), forced expiratory volume in 1 s (MD = 0.76, 95%CI: 0.41–1.11), functional capacity (SMD = 0.61, 95%CI: 0.08–1.13), while inspiratory muscle training could improve maximal inspiratory pressure (MD = 11.60, 95%CI: 8.15–15.05), maximal expiratory pressure (MD = 7.06, 95%CI: 3.50–10.62), forced vital capacity (MD = 0.71, 95%CI: 0.21–1.21), peak expiratory flow (MD = 0.84, 95%CI: 0.37–1.31), forced expiratory volume in 1 s (MD = 0.40, 95%CI: 0.08–0.72).

**Conclusions:**

This study provides good-quality evidence that respiratory muscle training is effective in improving respiratory muscle strength, pulmonary function, and functional capacity for patients with early stroke. Inspiratory muscle training combined with expiratory muscle training seems to promote functional recovery in patients with early stroke more than inspiratory muscle training alone.

**Trial registration:**

Prospero registration number: CRD42021291918.

**Supplementary Information:**

The online version contains supplementary material available at 10.1186/s11556-024-00338-7.

## Introduction

Stroke has become a major global health problem due to its high incidence, high disability rate, high recurrence rate, high mortality rate and high cost [1–3]. With the improvement of medical management for early stroke, most early stroke patients can survive but face loss of function [3–5]. Early stroke not only affects sensory, motor, cognitive and verbal functions, but also affects respiratory function [6, 7]. However, people usually give priority to the recovery of limb dysfunction in patients with early stroke and pay less attention to respiratory dysfunction after early stroke [8]. Although there is no precise report on the incidence of respiratory dysfunction in early stroke patients, previous studies have found that 18–88% of stroke patients have respiratory dysfunction [6, 9]. Research shows that stroke recovery usually occurs within three months of stroke onset, while 15–30% of stroke patients may have permanent dysfunction after 3 months of onset [6, 10, 11]. This means that the critical period for respiratory function recovery in early stroke patients may be within three months after the onset of stroke. The study would pay attention to respiratory dysfunction in people who had a stroke within three months.

Respiratory dysfunction in patients with early stroke may be related to respiratory centre damage after stroke [9, 12]. Stroke lesions can induce a series of pathophysiological reactions like inflammation, oxidative stress, metabolic abnormalities, excitatory toxicity and apoptosis, which can affect the respiratory centre, destroy the nerve conduction pathways related to respiration, and further damage the integration and regulation ability of respiratory-related sensory input, thereby reducing the activity function of respiratory muscle and eventually leading to respiratory dysfunction [13]. Besides, patients may suffer from malnutrition, secondary pain, reduced hemiplegic side activity, abnormal muscle tension, chest contracture, and even immobilization after early stroke, which may cause secondary dysfunction such as respiratory pattern disorder, insufficient pulmonary ventilation and gas exchange, decreased respiratory muscle contraction coordination, abnormal thoracic activity, and ultimately further lead to respiratory dysfunction [9, 14–16]. Respiratory dysfunction can increase the mortality rate of early stroke patients by 2–6 times, prolong the average hospitalization time, aggravate neurological dysfunction and lose their self-care ability [17, 18]. Therefore, it is necessary to explore effective respiratory rehabilitation to improve the respiratory function of early stroke patients and promote functional recovery.

Respiratory muscle training (RMT) is a kind of continuous and standardized training of inspiratory muscles or expiratory muscles, which increases the strength and endurance of respiratory muscles by improving maximum inspiratory pressure (MIP) and maximum expiratory pressure (MEP) [19]. The conventional approach of RMT is to perform repetitive breathing exercises with a hand-held respiratory training device to provide a pressure threshold or flow-dependent resistance against inhalation or exhalation to stimulate the respiratory musculature to respond and produce changes in muscle structure [19, 20]. Clinical studies suggest that RMT may be beneficial to the recovery of stroke patients [21, 22]. However, evidence for its effect on early stroke patients remains unclear. Therefore, available clinical studies need to be reviewed for the effects of RMT in early stroke patients.

In recent years, systematic reviews and meta-analyses studying the effects of RMT in stroke patients have increased. Seven systematic reviews collated evidence that RMT can improve respiratory function in stroke [17, 23–28]. Five studies reported that RMT can improve exercise tolerance in stroke patients [17, 23–25, 27]. Pollock et al. [29] pointed out that RMT may improve respiratory function in stroke patients but further research is needed. While Xiao et al. [8] held a different result that there was insufficient evidence to support the effect of RMT on post-stroke function. However, these systematic reviews did not separately study the efficacy of RMT in early stroke patients, and the studies included in these systematic reviews mixed early and chronic stroke patients. Therefore, an updated review for RMT in patients with early stroke of the existing literature is required. The review was the first-time analysis of the early stroke stage.

Thus, the objectives of this review were to examine the effects of RMT on respiratory function and functional capacity in patients with early stroke.

## Materials and methods

This meta-analysis was conducted following the preferred reporting items for systematic reviews and meta-analysis (PRISMA) guidelines [30].

### Eligibility criteria

The inclusion criteria were made (as detailed in Table 1) according to the Population-Interventions-Comparison-Outcomes of interest-Study design (PICOS) framework. The exclusion criteria were: (1) unusable full text, abstract-only papers, or protocol; (2) stroke with congestive heart failure; (3) insufficient data for effect size (ES) and 95% confidence interval (CI); (4) inappropriate intervention methods, for example, the description of the training program is unclear about the intensity, duration and frequency of the training; (5) studies with less than 4 points of the PEDro [26, 27, 31, 32].

**Table 1:** Inclusion criteria

**Study design**: RCTs in any language
**Population:** Stroke patients with age ≥ 18 years old. Early stroke < 3 months at the time of diagnosis
**Intervention:** Respiratory muscle training, including inspiratory and expiratory muscle training
**Control:** Sham respiratory muscle training (without any resistance) or regular rehabilitation programs without respiratory muscle training
**Outcomes:** 1. Respiratory function: respiratory muscle strength (MIP, MEP), respiratory muscle endurance, pulmonary function testing (PEF, FEV1, FVC), and dyspnea fatigue score. 2. Functional capacity: 6-min walking test, Fugl-Meyer Assessment, Functional Ambulation Category

*Key*: *RCTs* Randomised Controlled Trials, *MIP* Maximal Inspiratory Pressure, *MEP* Maximal Expiratory Pressure, *PEF* Peak Expiratory Flow, *FEV1* Forced Expiratory Volume in 1 s, *FVC* Forced Vital Capacity

### Information sources

The search was conducted in PubMed, Embase, PEDro, ScienceDirect, AMED, CINAHL, and China National Knowledge Infrastructure databases from inception to December 8, 2023.

### Search strategy

Keywords and associated terms were used flexibly in the retrieval process, combined with boolean operators and truncations, to ensure that the retrieved literature is related to the subject. The language was not limited in the actual retrieval process to reduce the deviation, although the language was restricted to English at the beginning of PROSPERO registration. A comprehensive and structured retrieval strategy was formulated as follows according to relevant retrieval guidelines. After the main database search, a further manual search was done from the reference list of all retained articles to ensure that comprehensive and complete literature can be retrieved. The specific search processes of all databases are shown in Additional file 1.

(“respiratory strength training” OR “inspiratory strength training” OR “expiratory strength training” OR “respiratory muscle training” OR “RMT” OR “inspiratory muscle training” OR “IMT” OR “expiratory muscle training” OR “EMT” OR “breathing muscle training” OR breathing exercises) AND (“acute stroke” OR “sub-acute stroke” OR “early stroke” OR “cerebrovascular accident” OR “stroke” OR “cerebral stroke” OR “CVA”) AND (“respiratory function” OR “respiratory muscle strength” OR “maximum inspiratory pressure” OR “MIP” OR “maximum expiratory pressure” OR “MEP” OR “respiratory muscle endurance” OR “pulmonary function testing” OR “peak expiratory flow” OR “PEF” OR “forced expiratory volume in 1 s” OR “FEV1” OR “forced vital capacity” OR “FVC” OR “dyspnea fatigue score” OR “functional capacity” OR “6-min walking test” OR “Fugl-Meyer assessment” OR “functional ambulation category”) AND (random* control* trials)

### Selection process

The retrieved studies were aggregated and stored in Endnote 20 software. After duplicate studies were removed, two reviewers (YS and JM) read the titles and abstracts of the remaining studies separately according to the inclusion and exclusion criteria, excluded the literature that did not meet the inclusion criteria, and then read the full texts of the literature that might meet the inclusion criteria to further judge whether they were included. Finally, the reviewers conducted face-to-face communication and proofreading of the final included studies. If the two reviewers disagree with the results of a study or eventual inclusion, it would be solved through discussion or consultation with a third reviewer (CS).

### Data collection process

Two reviewers (YS and LH) performed independently data extraction related to the evaluation question using standard data extraction forms adapted from the Joanna Briggs Institute (JBI) tool due to its ease in collecting and presenting relevant data, allowing for an effortless comparison and analysis between each of the studies [33, 34]. To ensure that relevant data was found and extracted while minimizing biases and other errors, the standard data extraction form has been tested before formal data extraction. The data extracted from the included articles were as follows: research background (author, publication year and country), research design, participant characteristics, sample size, intervention details (modality, intensity, training time, duration, device), control group management, outcome measures, and results (mean and standard deviation of outcomes) according to the suggestion of "Cochrane Handbook for Systematic Reviews of Interventions" [35]. When there were a lack of relevant data or problems in this process, the reviewer (JH) contacted the corresponding author to obtain relevant information. The extracted data were verified by a third reviewer (KZ).

### Methodological quality and risk of bias assessment

The tool chosen for the quality appraisal of this meta-analysis was the PEDro scale and cut-off values for PEDro scores were considered when selecting studies. Since the PEDro scale is an effective and reliable scoring tool for evaluating methodological quality within the physiotherapy profession and has been used frequently in systematic reviews and meta-analysis [35, 36]. The PEDro scale includes 11 items, including one external validity (eligibility criteria and source), eight items assessing the risk of bias (random allocation, concealed allocation, baseline comparability, blinding of participants, blinding of therapists, blinding of assessors, adequate follow-up (> 85%), intention-to-treat analysis), and two items assessing the completeness of the statistical report on the risk of bias (between-group statistical comparisons, reporting of point measures and measures of variability) [36, 37]. The total score ranges from 0 to 10 (the first item is not included), and higher scores indicate superior methodological quality [36]. Studies with scores between 9 and 10 are considered ‘excellent’, and scores from 6 to 8 are assessed as good, whereas scores of 5 and 4 are classified as fair quality, and scores below 4 are considered poor quality [38, 39]. Additionally, the risk of bias in included studies was assessed using the items of the Cochrane Risk of Bias tool and recorded in Review Manager 5.4 [35]. Two reviewers (YS and JX) with the same critical evaluation knowledge level used the PEDro score and the Cochrane Risk of Bias tool to independently assess methodological quality and risk of bias for included studies. Any disagreements with the score were resolved through discussion. If there were still any disagreements between the two primary reviewers, a third reviewer (YD) would resolve them.

### Data synthesis and analysis

RevMan 5.4. was used for data synthesis and analysis [35]. Meta-analysis was conducted only when the data of the analyzed variables were at least 3 studies. The heterogeneity among the studies was evaluated by the Cochrane Q statistic and the inconsistency index (I^2^) interpreted according to the Cochrane methodology [35]. The statistical heterogeneity was categorized as negligible or small heterogeneity (0 − 40%); moderate heterogeneity (30 − 60%); substantial heterogeneity (50 − 90%); and large/large heterogeneity (> 75%) [40]. The study was considered heterogeneous if the Cochran’s Q statistic tested for significance (*p* < 0.1) or the I^2^ was > 50% [41]. Since the use of the Q statistic is questioned when the number of included studies in the meta-analysis is small and the within-study variance is large, a random-effects model was used in this study. The separate pooled estimate of ES and their respective 95% CI was calculated, and the difference was significant when the test level was *p* < 0.05. If the standard deviation (SD) of change was not available, meta-analyses were performed using the standard deviation of baseline measurements. In addition, when the mean and SD were not present and the available statistics were the median and quartile range (IQR), these estimates of CI were transformed using IQR divided by 1.35.

Subgroup analyses were performed to assess the effect of different types of RMT (IMT + EMT or IMT only) on outcome variables in patients with early stroke if there were a sufficient number of studies. Sensitivity analyses were performed by removing studies one by one to assess the robustness or reliability of the pooled results for each variable, and to detect whether any studies produced significant heterogeneity between RMT and ES pooled estimations. Publication bias and other types of information bias in the meta-analysis would be comprehensively tested using funnel plots if the included studies were at least 10 or more [35, 42].

## Results

**Fig. 1:**
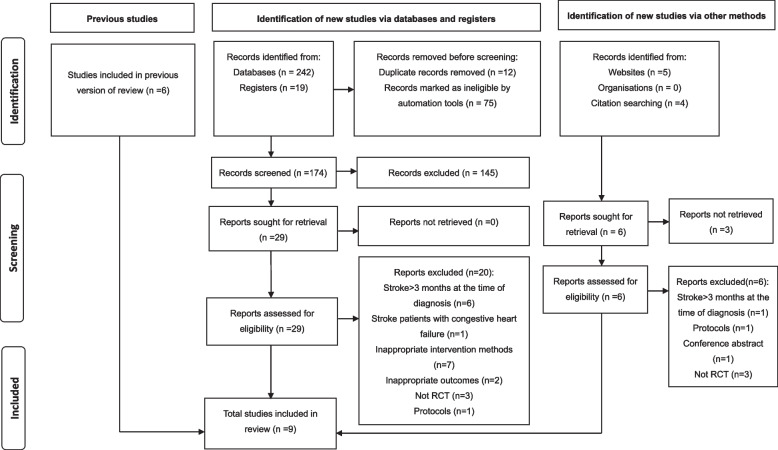
PRISMA search flow diagram

A total of 270 studies were retrieved, including 242 studies from databases, 19 studies from registers, and 9 studies from websites and citation searching. After excluding 87 duplicate studies, titles and abstracts were read, 145 irrelevant articles were excluded, and the full texts of the remaining studies were sought for reading and eligibility assessment. Finally, 35 studies were evaluated for inclusion, of which 26 studies were excluded for reasons (like stroke more than 3 months at the time of diagnosis, inappropriate intervention methods, not RCT), and 9 studies met the eligibility criteria and were included for quality analysis. Figure 1 shows the PRISMA flow diagram of study selection.

### Characteristics of the included studies

The included 9 studies were conducted between 2015 and 2022, while 3 studies [6, 43, 44] published in 2021 and 2022 updated the topic of this section and were not included in the previous review. Studies were conducted in China [43–46], Korea [6, 47], Spain [22, 48], and Britain [49]. Although the included studies varied by location, the synthesis of evidence can provide guidance and help for practice and further research. The main characteristics of the included studies are summarized in Table 2.

**Table 2:** Characteristics of the included 9 studies

Study	Country	Participants	Intervention	Control	Outcome measures
Liu et al. 2022 [44]	China	*n* = 86 IG: 46 CG: 40 65% men 51 years Stroke < 3 months MIP: NR MEP: NR	Modality: IMT + EMT Intensity: 30% of MIP and MEP Frequency: 30 min, 6 times/week Duration: 6 weeks Device: threshold resistance (Saike (Xiamen) Medical Equipment Co., Ltd) Progression: NR Regular rehabilitation programs as CG	Regular rehabilitation programs: limb stretching training, rollover training, balance training (sitting and standing), and gait training (30 min, 6 times/week, 6 weeks)	Respiratory function: FVC, FEV1, PEF Functional capacity: FMA
Choi et al. 2021 [6]	Korea	*n* = 44 IG: 22 CG: 22 47.7% men 67.4 years Stroke < 2 weeks MIP: NR MEP: NR	Modality: IMT + EMT Intensity: 50% of MIP and MEP Frequency: 30 min, 5 times/week Duration: 4 weeks Device: POWERbreathe (POWERbreathe International Ltd) and Threshold IMT/PEP (variable resistance) (Philips Respironics) Progression: resistance increased 1 or 2 cmH_2_O as the participants became accustomed to the resistance Regular rehabilitation programs as CG	Regular rehabilitation programs: physical and occupational therapy sessions (5 times/week, 4 weeks)	Respiratory function: MIP, MEP, FVC, FEV1, PEF Functional capacity: FAC
Sun et al. 2021 [43]	China	*n* = 50 IG: 25 CG: 25 54% men 50.4 years Stroke < 3 months MIP: NR MEP: NR	Modality: IMT + EMT Intensity: 30% of MIP and MEP Frequency: 30 min, 6 times/week Duration: 6 weeks Device: threshold resistance (Saike (Xiamen) Medical Equipment Co., Ltd) Progression: NR Regular rehabilitation programs as CG	Regular rehabilitation programs: physical factor therapy, muscle strength training, stretching training, balance training (sitting and standing), gait training, and activity of daily living training (30 min, 6 times/week, 6 weeks)	Respiratory function: FVC, FEV1, PEF Functional capacity: FMA
Gu et al. 2020 [45]	China	*n* = 60 IG: 30 CG: 30 38.3% men 59.3 years Stroke < 2 weeks MIP: NR MEP: NR	Modality: MIP Intensity: 30% of MIP Frequency: 5–10 sets of 3 repetitions, 5 times/week Duration: 8 weeks Device: SECCO S2 intelligent respiratory training instrument (variable resistance) ( Saike (Xiamen) Medical Equipment Co., Ltd) Progression: resistance increased the pressure by 10% after 3 days and gradually increased the training load according to the participant’s tolerance Regular rehabilitation programs as CG	Regular rehabilitation programs: good limb placement, posture management, exercise therapy, occupational therapy, acupuncture, low and medium frequency electrotherapy (once a day, 5 times/week, 8 weeks)	Respiratory function: MIP, MEP, FVC, PEF
Yoo and Pyun 2018 [47]	Korea	*n* = 40 IG: 20 CG: 20 65% men 61 years Stroke < 3 months MIP: NR MEP: NR	Modality: IMT + EMT Intensity: NR Frequency: 30 min, twice a day, 7 times/week Duration: 3 weeks Device: A flow-oriented incentive spirometer for IMT (Hyupsung, Seoul, Korea); Acapella vibratory PEP for EMT (threshold resistance) (Smith Medical, Minnesota, MN, USA) Progression: NR Regular rehabilitation programs as CG	Regular rehabilitation programs: motion exercises, muscle strengthening, gait training, fine motor exercises, and activity of daily living training (30 min, twice a day, 5 days/week, 3 weeks)	Respiratory function: FVC, FEV1, PEF Functional capacity: FMA
Guillén-Solà et al. 2017 [22]	Spain	*n* = 41 IG: 20 CG: 21 68.3% men 68.4 years Stroke within 1 to 3 weeks MIP: 37 cmH_2_O MEP: 56 cmH_2_O	Modality: IMT + EMT Intensity: 30% of MIP and MEP Frequency: 5 sets of 10 repetitions, twice a day, 5 times/week Duration: 3 weeks Device: Orygen Dual Valve (threshold resistance) (Orygen Dual Valve®, Forumed SL, Barcelona, Catalonia, Spain) Progression: resistance increased weekly at intervals of 10 cmH_2_O Regular rehabilitation programs as CG	Regular rehabilitation programs: physical, occupational and speech therapy targeting specific impairments in mobility, activities of daily living, standard swallow therapy and communication skills (3 h per day, 5 times/week, 3 weeks) Sham MIP + MEP with the workloads fixed at 10 cmH_2_O (5 sets of 10 repetitions, twice a day, 5 times/week, 3 weeks)	Respiratory function: MIP, MEP
Yu et al. 2016 [46]	China	*n* = 45 IG: 22 CG: 23 52.7% men 66.4 years Stroke within 1 to 3 weeks MIP: NR MEP: NR	Modality: IMT Intensity: 30% of MIP Frequency: 20–30 min, twice a day, 6 days/week Duration: 3 weeks Device: Threshold IMT (threshold resistance) (Yuyao Shengchang Medical Equipment Co., Ltd) Progression: resistance increased the training load by 10% Regular rehabilitation programs as CG	Regular rehabilitation programs: nerve stimulation, muscle strength training, balance function training, gait training, low and medium frequency electric therapy, acupuncture, and daily living ability training (twice a day, 6 days/week, 3 weeks)	Respiratory function: MIP, MEP, FVC, FEV1, PEF Functional capacity: FMA
Kulnik et al. 2015 [49]	Britain	*n* = 51 IG: 26 CG: 25 60.2% men 64.4 years Stroke < 2 weeks MIP: 42 cmH_2_O MEP: 61 cmH_2_O	Modality: IMT Intensity: 50% of MIP Frequency: 5 sets of 10 breaths/day, 7 times/week Duration: 4 weeks Device: Threshold IMT; Threshold PEP-Respironics (threshold resistance) (Respironics, Parsippany, NJ) Progression: resistance adjusted to 50% of MIP every week	Sham MIP + MEP with a fixed load of 10% of maximum pressure (5 sets of 10 breaths/day, 7 times/week, 4 weeks)	Respiratory function: MIP, MEP
Messaggi-Sartor et al. 2015 [48]	Spain	*n* = 109 IG: 56 CG: 53 57.8% men 66.5 years Stroke < 3 weeks MIP: 41 cmH_2_O MEP: 63 cmH_2_O	Modality: IMT + EMT Intensity: 30% of MIP and MEP Frequency: 5 sets of 10 repetitions, twice a day, 5 times/week Duration: 3 weeks Device: Orygen-Dual valve (threshold resistance) ( LungOn, European distributors) Progression: resistance increased 10 cmH_2_O every week Regular rehabilitation programs as CG	Regular rehabilitation programs: physical, occupational, and speech therapy sessions (3 h per day, 5 days a week, 3 weeks) Sham MIP + MEP at a fixed workload of 10 cmH_2_O (5 sets of 10 repetitions, twice a day, 5 times/week, 3 weeks)	Respiratory function: MIP, MEP

*Key*: *IG* Intervention Group, *CG* Control Group, *RMT* Respiratory muscle training, *IMT* Inspiratory muscle training, *EMT* Expiratory muscle training, *MIP* Maximal Inspiratory Pressure, *MEP* Maximal Expiratory Pressure, *FVC* Forced Vital Capacity, *FEV1* Forced Expiratory Volume in 1 s, *PEF* Peak Expiratory Flow, *FMA* Fugl-Meyer Assessment, *FAC* Functional Ambulation Category, *NR* not reported

#### Participants

The 9 studies enrolled 526 participants, with the number of participants varying from 40 [47] to 109 [48]. The mean age of the participants was 61.6 years. Participants included in all studies contained both genders, but there was an overall predominance of male participants. All participants in this study had less than three months of stroke onset. Three studies [6, 45, 49] included patients within 2 weeks of stroke. Two studies [22, 46] included patients within 1 to 3 weeks of stroke. One study [48] included patients within 3 weeks of stroke. Others [43, 44, 47] included patients within 3 months of stroke. Most included studies did not assess the initial MIP and MEP, while only 3 studies [22, 48, 49] evaluated the initial MIP and MEP. The average of the initial MIP and MEP was 40 cmH_2_O and 60 cmH_2_O respectively. Research shows that the normal values of MIP and MEP are as follows: MIP (118.4 ± 37.2 cmH_2_O for men, 84.5 ± 30.3 cmH_2_O for women), MEP (140 ± 30cmH_2_O for men, 95 ± 20 cmH_2_O for women), while lower than normal values are considered a decrease in MIP and MEP [50, 51]. The original average MIP and MEP in the included studies are both considered decreases.

#### Interventions

All included studies performed RMT in the intervention group (IG). Six studies performed IMT and EMT [6, 22, 43, 44, 47, 48], while the remaining studies only had IMT [45, 46, 49]. Both IG and the Control Group (CG) carried out regular rehabilitation therapy. Six studies [6, 43–47] used regular rehabilitation programs in the CG. Three studies [22, 48, 49] used sham MIP + MEP with the workloads fixed in the CG, which was not enough to improve respiratory muscle strength or endurance. Regarding the devices used in the IG, they were different: Threshold, Orygen-Dual valve, Respironics, A flow-oriented incentive spirometer, Acapella vibratory, SECCO S2 intelligent respiratory training instrument, POWERbreathe, all from the manufacturer (Details in Table 2). Although different types of devices were used, most of the studies were threshold resistance, while only Gu et al. [45] and Choi et al. [6] were variable resistance.

The parameters of intervention were different across the studies. Although the intensity of RMT in the included studies varied, they started at 30% to 50% of MIP/MEP and were adjusted with the intervention weekly or biweekly. RMT with an intensity of less than 30% may not improve inspiratory muscle strength and exercise tolerance [27]. Additionally, the frequency and duration also varied in these studies. The time of the sessions varied from 20 to 30 min. Five studies [6, 43, 44, 46, 47] tended to adopt 30 min, and the remaining studies [22, 45, 48, 49] used 5 sets or 5–10 sets with 3 or 10 repetitions. Furthermore, sessions were carried out 5 to 14 times per week. The duration of the intervention ranged between 3 to 8 weeks.

### Methodological quality and risk of bias of included studies

**Fig. 2:**
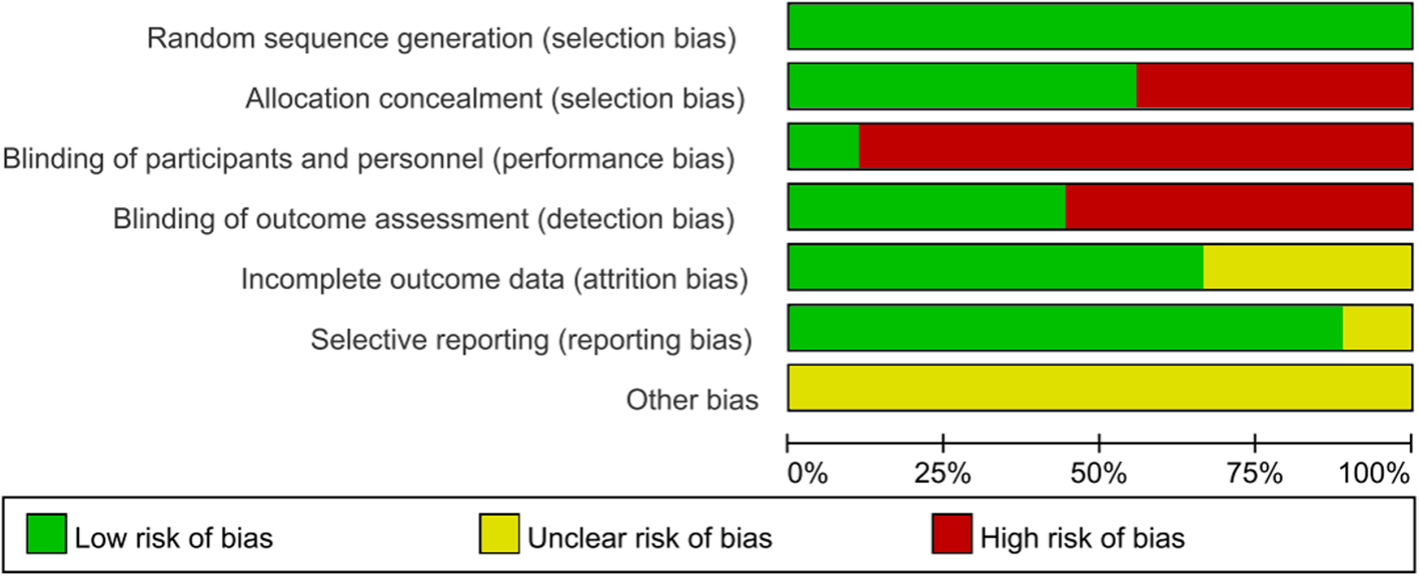
Risk of bias graph the included 9 studies

The methodological quality and risk of bias of included studies were critically evaluated using the PEDro scale and the Cochrane Risk of Bias tool. Table 3 presents each item score and the results of 9 RCT studies. The risk of bias graph is shown in Fig. 2. The studies of Messaggi-Sartor et al. [48] and Yu et al. [46] were the highest scores with a PEDro score of eight points, and their methodological quality can be regarded as 'good'. Furthermore, there were also 6 studies [6, 22, 43–45, 49] whose methodological quality can be regarded as 'good'. Though the lowest score was five points for the study of Yoo and Pyun [47], its methodological quality can be regarded as ‘fair’. The mean PEDro score of the included studies was 6.5 (range 5 to 8). Therefore, it is considered good-quality evidence.

**Table 3:** Study quality on the PEDro Scale of 9 studies

Study	Random allocation	Concealed allocation	Baseline similarity	Blind subjects	Blind therapists	Blind assessors	Adequate follow-up	Intention-to-threat analysis	Between-group comparisons	Point estimates and variability	Total score
Liu et al. 2022 [44]	Y	Y	Y	N	N	N	Y	Y	Y	Y	7
Choi et al. 2021 [6]	Y	Y	Y	N	N	N	N	Y	Y	Y	6
Sun et al. 2021 [43]	Y	N	Y	N	N	N	Y	Y	Y	Y	6
Gu et al. 2020 [45]	Y	N	Y	N	N	N	Y	Y	Y	Y	6
Yoo and Pyun 2018 [47]	Y	N	Y	N	N	N	Y	N	Y	Y	5
Guillén-Solà et al. 2017 [22]	Y	N	Y	N	N	Y	N	Y	Y	Y	6
Yu et al. 2016 [46]	Y	Y	Y	N	N	Y	Y	Y	Y	Y	8
Kulnik et al. 2015 [49]	Y	Y	Y	N	N	Y	N	Y	Y	Y	7
Messaggi-Sartor et al. 2015 [48]	Y	Y	Y	Y	N	Y	N	Y	Y	Y	8

*PEDro* Physiotherapy Evidence Database, *Y* Yes, *N* No. The total score of PEDro: 10

All the critically appraised studies reported random allocation, baseline similarity, between-group differences, and point estimate and variability. Eight studies [6, 22, 43–46, 48, 49] provided intention-to-treat analysis. Five studies reported respectively appropriate follow-up data [43–47] and reported concealed allocation [6, 44, 46, 48, 49]. Four studies [22, 46, 48, 49] provided blindness to outcome assessors, which may introduce detection bias. However, only one studies [48] showed blind participants. None of the studies blinded the therapists. Participants and therapists were not blinded, which could lead to performance bias. However, performance bias should not be considered a preferential bias effect because it is difficult or impossible to blind participants and therapists in the process of implementing complex interventions [52]. Therefore, despite the performance bias and detection bias in these studies, the lack of blinding for participants and therapists was accepted in this review but taken into account when interpreting the results.

### Effect of interventions

#### Effect of respiratory muscle training on respiratory muscle strength

**Fig. 3:**
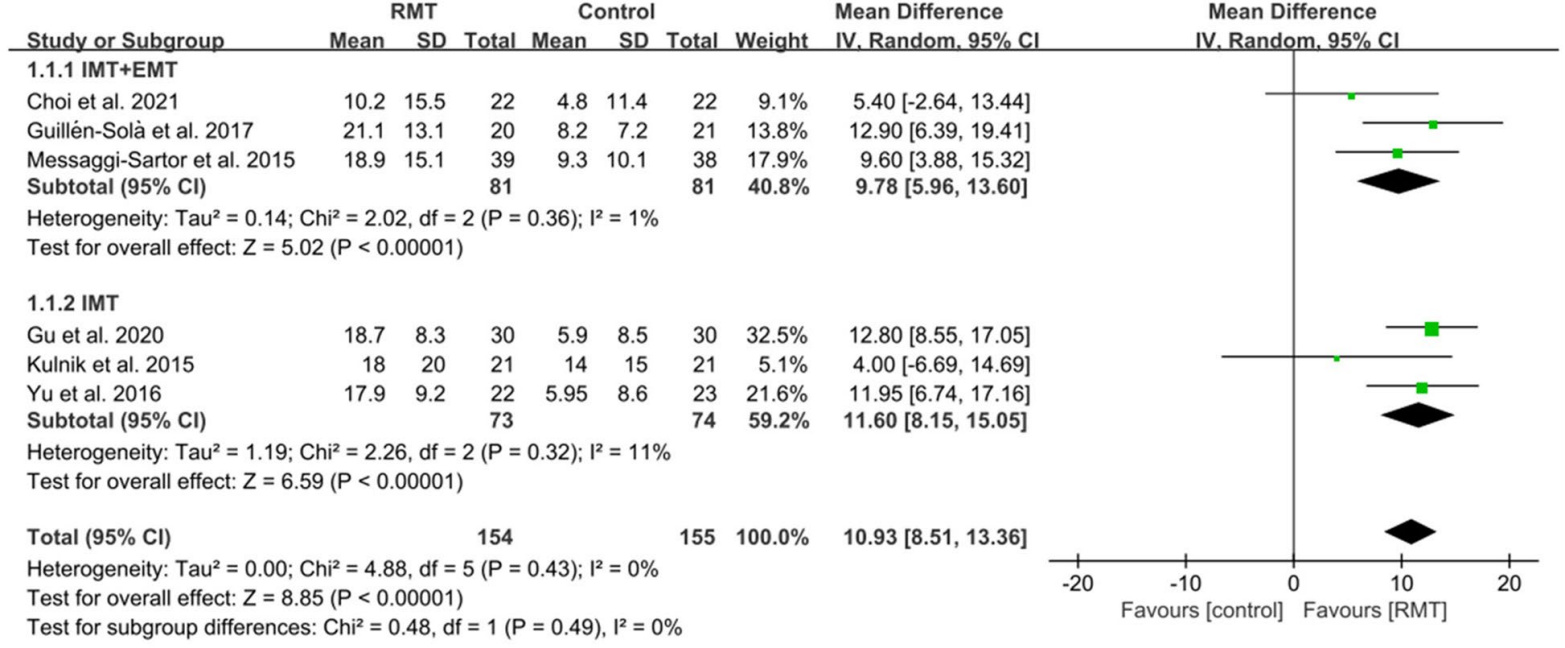
The pooled effect size of respiratory muscle training (RMT) on Maximal Inspiratory Pressure (MIP) between RMT and control groups. IMT = inspiratory muscle training; EMT = expiratory muscle training

**Fig. 4:**
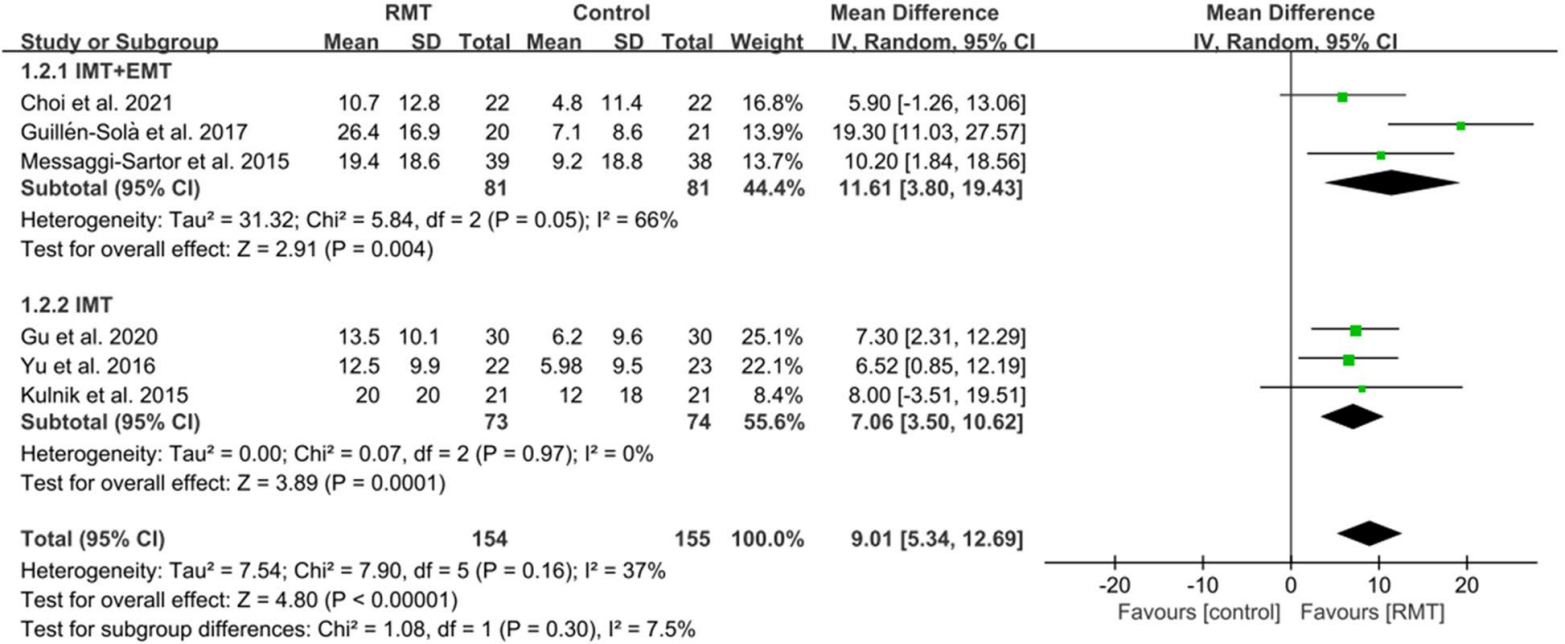
The pooled effect size of respiratory muscle training (RMT) on Maximal Expiratory Pressure (MEP) between RMT and control groups. IMT = inspiratory muscle training; EMT = expiratory muscle training

The MIP and MEP were assessed in six studies [6, 22, 45, 46, 48, 49]. RMT produced a statistically significant effect on improving MIP (*n* = 309, MD = 10.93, 95%CI: 8.51-13.36, *p* < 0.00001, I^2^ = 0%) (Fig. 3) and MEP (*n* = 309, MD = 9.01, 95%CI: 5.34–12.69, *p* < 0.00001, I^2^=37%) (Fig. 4) in patients with early stroke compared to the CG. Sensitivity exclusion analysis showed that no study significantly affected the pooled results of MIP and MEP after excluding the study one by one.

Three studies [6, 22, 48] performed IMT + EMT, while three studies [45, 46, 49] only carried out IMT. The subgroup analysis showed a statistically improvement in MIP for IMT + EMT (*n* = 162, MD = 9.78, 95%CI: 5.96-13.60, *p* ＜0.00001, I^2^ = 1%) and IMT (*n* = 147, MD = 11.60, 95%CI: 8.15–15.05, *p*＜0.00001, I^2^ = 11%) (Fig. 3). Similarly, the subgroup analysis also displayed a statistically increase in MEP for IMT + EMT (*n* = 162, MD = 11.61, 95%CI: 3.80–19.43, *p* = 0.004, I^2^ = 66%) and IMT (*n* = 147, MD = 7.06, 95%CI: 3.50–10.62, *p* = 0.0001, I^2^ = 0%) (Fig. 4). Sensitive exclusion analysis suggested that no study significantly affected the pooled results of MIP for IMT+EMT and IMT. However, the pooled result of MEP for IMT + EMT wasnot statistically significant through excluding the study of Messaggi-Sartor et al [48]. There was no statistically significant difference between IMT + EMT and IMT in improving MIP (*p* = 0.49) (Fig. 3) and MEP (*p* = 0.30) (Fig. 4).

#### Effect of respiratory muscle training on pulmonary function

**Fig. 5:**
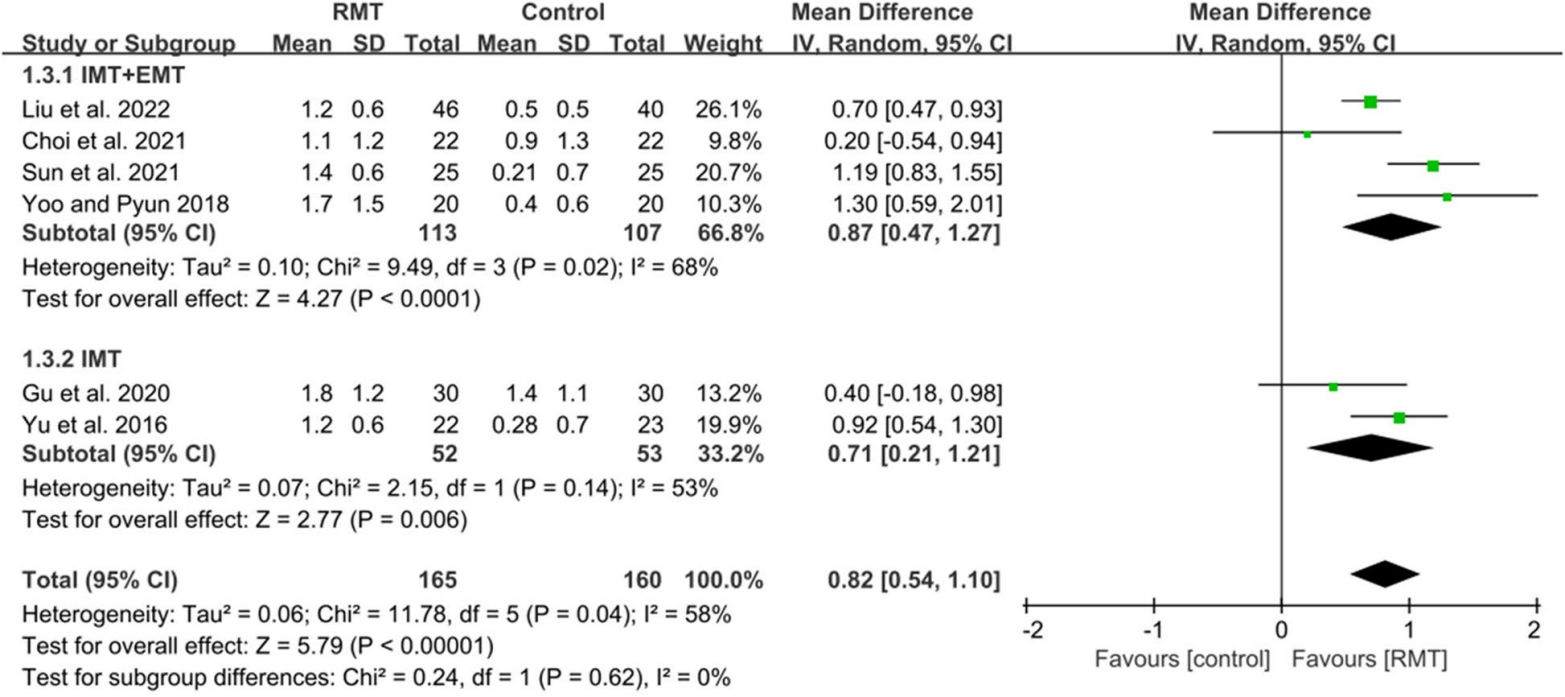
The pooled effect size of respiratory muscle training (RMT) on Forced Vital Capacity (FVC) between RMT and control groups. IMT = inspiratory muscle training; EMT = expiratory muscle training

**Fig. 6:**
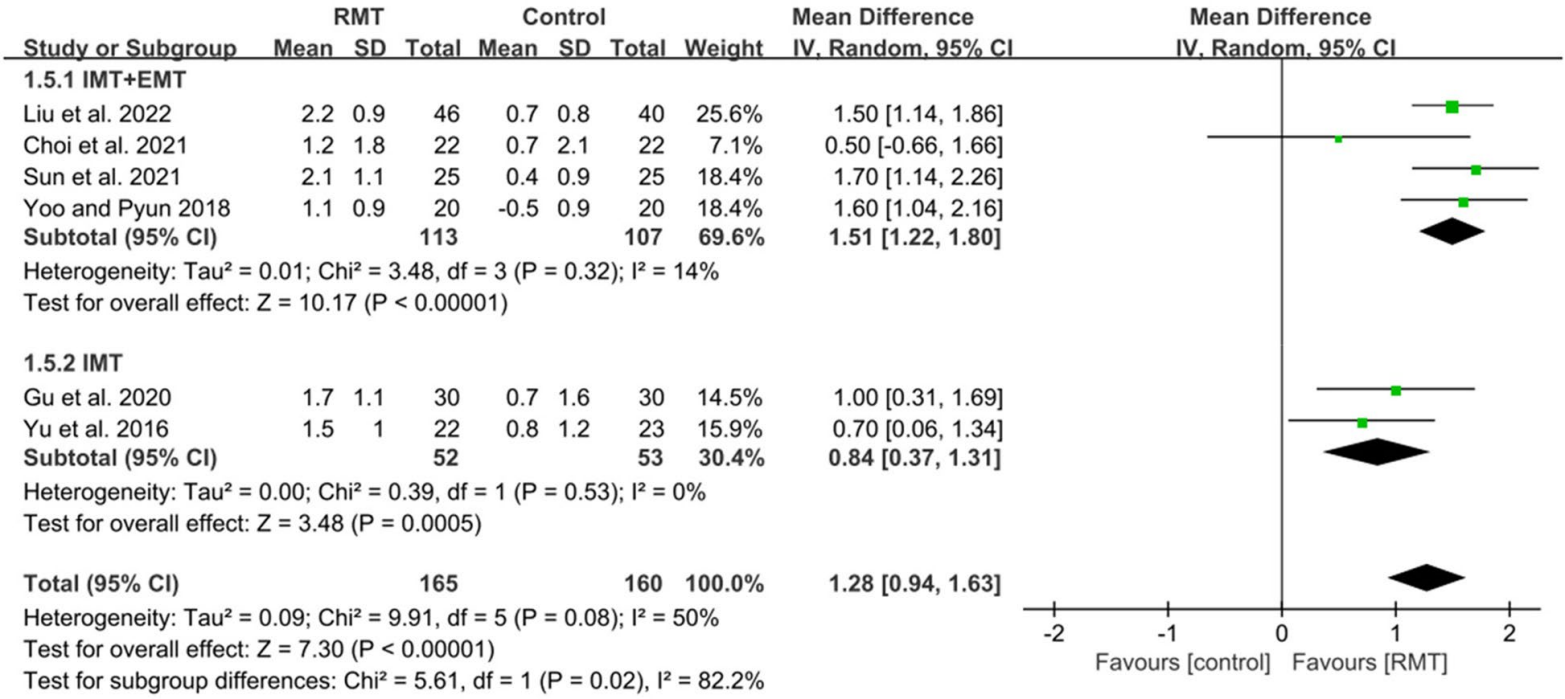
The pooled effect size of respiratory muscle training (RMT) on Peak Expiratory Flow (PEF) between RMT and control groups. IMT = inspiratory muscle training; EMT = expiratory muscle training

**Fig. 7:**
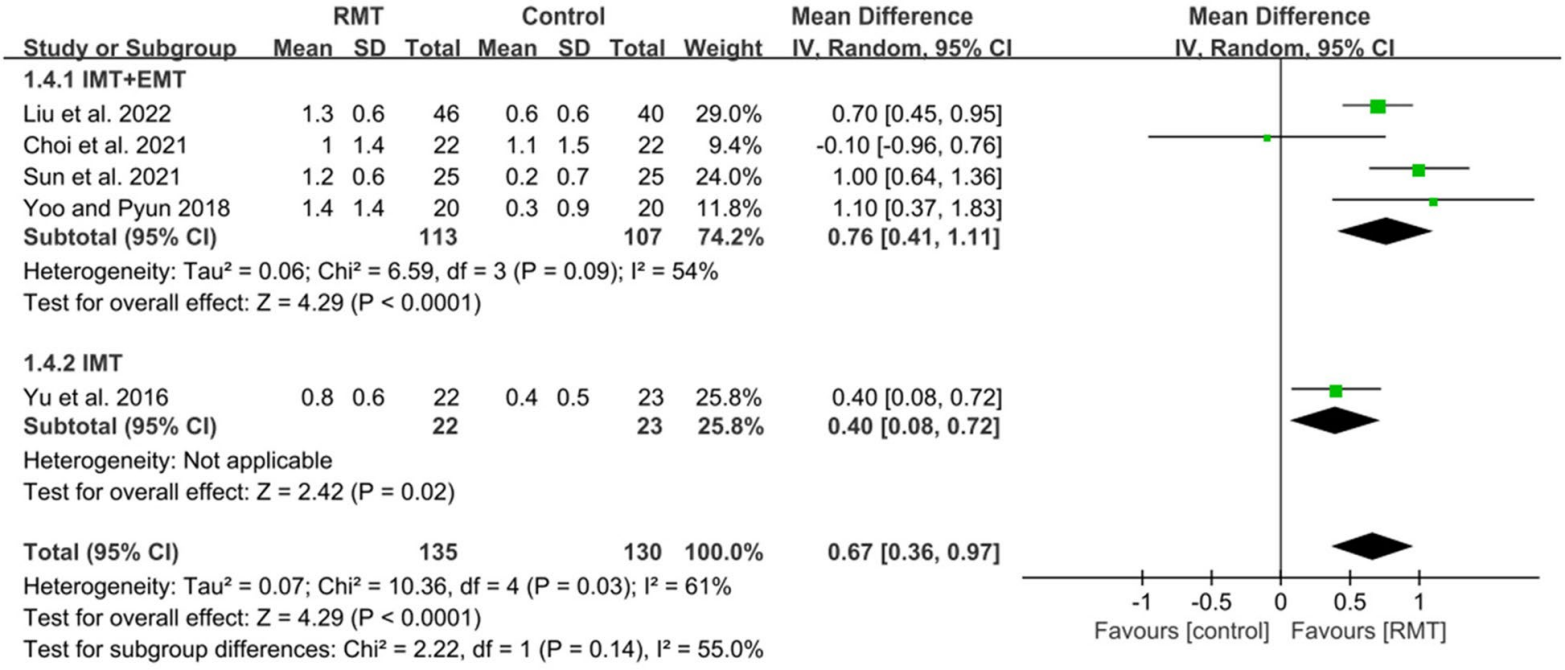
The pooled effect size of respiratory muscle training (RMT) on Forced Expiratory Volume in 1 s (FEV1) between RMT and control groups. IMT = inspiratory muscle training; EMT = expiratory muscle training

Six studies [6, 43–47] measured the results about pulmonary function. These studies all analyzed FVC and PEF. Five studies reported FEV1 except for the study of Gu et al [45]. The pooled data suggested that RMT produced a statistically significant increase in FVC (*n* = 325, MD = 0.82, 95%CI: 0.54–1.10, *p* < 0.00001, I^2^ = 58%) (Fig. 5), PEF (*n* = 325, MD = 1.28, 95%CI: 0.94–1.63, *p* < 0.00001, I^2^ = 50%) (Fig. 6) and FEV1 (*n* = 265, MD = 1.36, 95%CI: 1.13–1.59, *p* < 0.00001, I^2^ = 61%) (Fig. 7) for patients with early stroke compared to the CG. Sensitivity analysis for pulmonary function variables showed that no study significantly influenced the pooled results of pulmonary function when studies were removed one by one.

Four studies [6, 43, 44, 47] performed IMT + EMT for FVC, PEF and FEV1, while only one studies [46] carried out IMT for FEV1 and two studies [45, 46] conducted IMT for FVC and PEF. The subgroup analysis indicated that IMT + EMT had a statistically improvement in FVC (*n* = 220, MD = 0.87, 95%CI: 0.47–1.27, *p* < 0.0001, I^2^ = 68%) (Fig. 5), PEF (*n* = 220, MD = 1.51, 95%CI: 1.22–1.80, *p* < 0.00001, I^2^ = 14%) (Fig. 6) and FEV1 (*n* = 220, MD = 0.76, 95%CI: 0.41–1.11, *p* < 0.0001, I^2^ = 54%) (Fig. 7). Sensitive exclusion analysis indicated that no study significantly affected these results. The subgroup analysis also indicated that IMT produced a statistically increase in FVC (*n* = 105, MD = 0.71, 95%CI: 0.21–1.21, *p* = 0.006, I^2^ = 58%) (Fig. 5), PEF (*n* = 105, MD = 0.84, 95%CI: 0.37–1.31, *p* = 0.0005, I^2^ = 0%) (Fig. 6) and FEV1 (*n* = 45, MD = 0.40, 95%CI: 0.08–0.72, *p* = 0.02) (Fig. 7). Sensitive exclusion analysis suggested that no study significantly affected the pooled result of PEF. However, IMT did not produce a statistically significant improvement in FVC after excluding the study of Yu et al [46]. Additionally, sensitive exclusion analysis could not be conducted for FEV1 because of only one study. There was no statistically significant difference between IMT + EMT and IMT in improving FVC (*p* = 0.62) (Fig. 5) and FEV1 (*p* = 0.14) (Fig. 7), while there was a statistically significant difference between IMT + EMT and IMT in improving PEF (*p* = 0.02) (Fig. 6).

#### Effect of respiratory muscle training on functional capacity

**Fig. 8:**
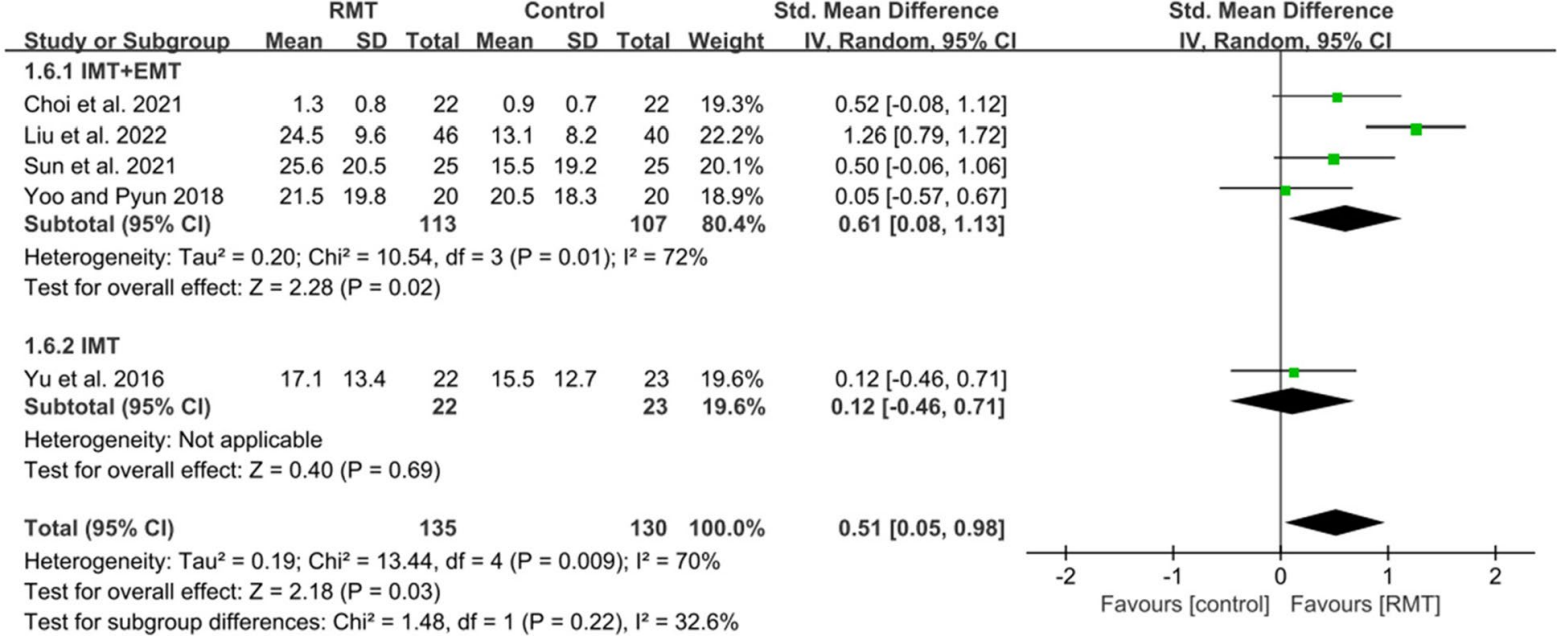
The pooled effect size of respiratory muscle training (RMT) on functional capacity between RMT and groups. IMT = inspiratory muscle training; EMT = expiratory muscle training

Five studies focused on functional capacity [6, 43, 44, 46, 47]. Four studies analyzed FMA [43, 44, 46, 47], while one study [6] assessed FAC. The meta-analysis was performed with SMD due to the differences between the assessment scales. RMT produced a statistically significant improvement in functional capacity for patients with early stroke compared to the CG (*n*=265, SMD=0.51, 95%CI: 0.05-0.98, *p*=0.009, I^2^=65.5%) (Fig. 8). Sensitive exclusion analysis showed that RMT did not have a statistically significant increase in functional capacity when the studies of Sun et al. [43] and Choi et al. [6]were excluded separately.

Four studies [6, 43, 44, 47] performed IMT + EMT, while only one studies [46] carried out IMT. The subgroup analysis showed that IMT + EMT had a statistically significant effect on improving functional capacity (*n* = 220, SMD = 0.61, 95%CI: 0.08–1.13, *p* = 0.01, I^2^ = 72) (Fig. 8). Sensitive exclusion analysis indicated that IMT + EMT did not produce a statistically significant improvement in functional capacity after excluding the studies of Sun et al. [43] and Choi et al. [6] respectively. On the contrary, IMT did not show a statistically significant improvement in motor function (*n* = 45, SMD = 0.12, 95%CI: -0.46–0.71, *p* = 0.69) (Fig. 8). Sensitive exclusion analysis could not be performed due to only one study. There was no statistically significant difference between IMT + EMT and IMT in improving functional capacity (*p* = 0.22) (Fig. 8).

## Discussion

This meta-analysis summarizes and analyzes the available evidence on the effects of RMT in patients with early stroke. This study provides good-quality evidence that RMT can improve respiratory muscle strength (MIP, MEP), pulmonary function (FVC, PEF, FEV1), and functional capacity in early stroke patients. Therefore, RMT can be considered an effective intervention for early-stage stroke patients.

Subgroup analysis showed that IMT + EMT was beneficial to the recovery of respiratory muscle strength (MIP and MEP), pulmonary function (FVC, PEF, FEV1), and functional capacity, while IMT could improve respiratory muscle strength (MIP and MEP) and pulmonary function (FVC, PEF, FEV1). Furthermore, subgroup analyses did not show significant differences between IMT + EMT and IMT interventions, except in PEF. IMT + EMT had a greater effect on PEF improvement than IMT alone. These seem to suggest that IMT + EMT can promote functional recovery in early stroke patients more than IMT alone. However, due to the limited number of studies on IMT + EMT and IMT when performing subgroup analysis, more RCTs are still needed to conduct research in this direction.

### Respiratory muscle strength

This study found that RMT significantly improved MIP and MEP in patients with early stroke, which is similar to the results of most previous reviews [17, 23–28]. However, Xiao et al. [8] found that there is insufficient evidence to support that RMT can improve respiratory muscle strength in stroke patients. This may be related to the insufficient literature included in their research and no separate study on early stroke patients. Moreover, IMT + EMT and IMT also improved MIP and MEP. Early stroke patients may have decreased respiratory muscle strength due to respiratory centre injury and central diaphragm dysfunction [12, 29, 53, 54], while RMT may improve the integration and regulation of respiratory-related sensory input by promoting the recovery of cerebral cortex respiratory centre [9, 53] and activating cortical spinal cord pathway [12, 55], thereby improving diaphragm function and respiratory muscle strength. Furthermore, RMT is more likely to change the adaptive structure of respiratory muscle [56] in patients with early stroke, thereby improving the strength of respiratory muscle. However, our previous study only found that RMT improved MIP in patients with chronic stroke without affecting MEP. This indicates that RMT may have different effects on stroke patients between early and chronic stages. This may be related to the easier recovery of central nervous system plasticity within three months [11], which means that RMT is more likely to promote the recovery of respiratory muscles in early stroke patients, while RMT may be relatively difficult to promote the improvement of the respiratory muscle in chronic stroke patients. Another possibility may be the limited number of studies investigating the effects of RMT on MEP in stroke patients between early and chronic stages. Therefore, more RCTs are still needed to study the effects of respiratory muscle training on MEP in stroke patients at different stages.

### Pulmonary function

The meta-analysis showed that RMT had a significant influence on FVC, PEF, and FEV1 in patients with early stroke. This finding is supported by the previous reviews [17, 23, 25, 26, 28]. Similarly, IMT + EMT and IMT also improved FVC, PEF, and FEV1. However, only PEF was different under the intervention of IMT + EMT and IMT. The effect of IMT + EMT on the improvement of PEF was greater than IMT alone. Early stroke patients may experience reduced the expansion of lung and chest wall, harden chest, reduce the compliance of chest wall, and change in the elastic properties of lung tissue because of respiratory muscle weakness, which can affect the lung volume, flow reduction and restrictive ventilation mode of early stroke patients [15, 27, 57–59]. RMT may improve pulmonary ventilation function by increasing respiratory muscle strength and thoracic expansion [13, 59, 60], inhibiting the weakening of lung tissue elasticity caused by limited mobility after early stroke [61–63]. Additionally, it may be because RMT has a pump effect on blood circulation [46], namely, diaphragm movement can promote the venous return to reduce pulmonary blood stasis, increase alveolar ventilation, and effectively exchange gas [64, 65]. However, our previous meta-analysis found that RMT did not improve FVC but improved PEF and FEV1 in chronic stroke patients. This may be related to several main reasons. The recovery of neurological function in chronic stroke patients is relatively stable [11, 66, 67], and therefore it may require the long duration and strong intensity of RMT to improve the muscle tension of the hemiplegic side chest wall and reduce spasms, thereby improving the elasticity of the lungs and thorax. Moreover, we also did not observe that RMT improved MEP during the chronic phase. It is possible that only the changes of MIP are not enough to improve the elastic recoil effect of lung tissue in the chronic phase, and thus no changes in FVC are observed. Compared with early stroke, because it is in the early stage of neurological recovery, RMT is more likely to improve respiratory muscle activity and the elasticity of the lung and thorax, thereby enhancing FVC, PEF, and FEV1.

### Functional capacity

The meta-analysis still found that RMT showed a positive effect on functional capacity in patients with early stroke. Previous systematic reviews [17, 23–25, 27] reported a similar result, although these studies mixed early and chronic stroke patients. Furthermore, IMT + EMT was helpful for functional capacity recovery, while IMT did not. However, this finding should be interpreted with caution due to the insufficient number of studies on IMT. The reduction of inspiratory muscle and expiratory muscle in early stroke patients can affect the recovery of motor function [68, 69]. Therefore, the improvement of inspiratory and expiratory muscle strength is important for functional ability in early stroke patients. RMT can enhance the oxygen-carrying capacity of respiratory muscle by increasing the respiratory muscle strength and lung function, and improve indirectly the exercise tolerance and function of early stroke patients [70–72]. However, in our previous study, we did not find any effect of RMT on functional abilities in patients with chronic stroke. This may be because the neurological function of patients with chronic stroke recovers slowly, and the duration and intensity of RMT are not sufficient to improve motor function [66, 67, 73]. Moreover, compared with the early stage, stroke patients at this stage are relatively less motivated [74, 75]. Another possibility is related to the limited number of included high-quality RCTs. Therefore, more RCTs are needed to explore the effects of RMT and different modalities of RMT on motor function in patients with early and chronic stroke.

### Clinical implications and study limitations

The currently available evidence seems to support the use of RMT in early stroke patients. Therefore, it is recommended to perform RMT as soon as possible for early stroke, so as to promote respiratory function and motor function. IMT + EMT combined training can be considered when performing RMT to achieve maximum functional recovery.

However, other aspects of this evidence still need to be considered in clinical practice. Firstly, the nine studies included were primarily placed in China, Korea, Spain, and Britain. Differences in stroke disability rates, participant demographics, medical level, and degree of patient rehabilitation cooperation across countries may reduce the generality of the findings, which should be considered in clinical use. Besides, given the limited number of high-quality studies available, only 9 RCTs were extracted and synthesized in this study. Not all studies reported the same variables. Therefore, the total number of participants included in each variable of the study may be insufficient, which can lead to minor trial bias. When performing subgroup analyses, partial results should be considered with caution as in some cases only 1 or 2 studies were included.

Moreover, only three studies reported the initial MIP and MEP, while patients with respiratory muscle weakness may generally respond better. Most of the included studies conducted RMT intervention based on conventional rehabilitation treatment, and increased overall activity levels may cause the superposition of treatment effects. These can affect the validity of the results. Additionally, the included studies were heterogeneous in intervention details, duration of intervention, and outcome collection, which may limit the strength of the synthesis of results. Finally, several interesting variables like respiratory muscle endurance, dyspnea fatigue, and walking ability were not present in the included studies, and RMT may help improve these variables in patients with early stroke. Thus, more clinical trials are needed to investigate whether RMT has an effect on these indicators in patients with early stroke.

## Conclusions

This study provides good-quality evidence that RMT is effective in improving respiratory muscle strength (MIP, MEP), pulmonary function (FVC, PEF, FEV1), and functional capacity for patients with early stroke. For different RMT modalities, IMT + EMT seems to promote functional recovery in patients with early stroke more than IMT alone. High-quality, large-scale RCTs are needed to study the most appropriate characteristics of RMT (like IMT and EMT alone or combined, optimal dose, duration and outcome) in patients with early stroke to achieve the best clinical curative effect.

### Supplementary Information

**Additional file 1. **Database search.

## Data Availability

Not applicable.

## Abbreviations

RCTs: Randomised control trials
PEDro: The physical therapy evidence database
PICOS: Population-Interventions-Comparison-Outcomes of interest-Study design
IG: Intervention Group
CG: Control Group
RMT: Respiratory muscle training
IMT: Inspiratory muscle training
EMT: Expiratory muscle training
MIP: Maximal Inspiratory Pressure
MEP: Maximal Expiratory Pressure
PEP: Positive Expiratory Pressure
FVC: Forced Vital Capacity
FEV1: Forced Expiratory Volume in 1 s
FIM: Functional independence measure
FAC: Functional Ambulation Category
MD: Mean differences
SMD: Standard mean differences
95%CI: 95% Confidence interval
JBI: The Joanna Briggs Institute
NR: Not reported
