# Supplementary material for: The effects of respiratory muscle training on respiratory function and functional capacity in patients with early stroke: a meta-analysis
Source: Eur Rev Aging Phys Act. 2024 Feb 22;21:4. doi: 10.1186/s11556-024-00338-7 (PMC10882726; doi:10.1186/s11556-024-00338-7)
Supplement: Supplementary file 1 — Additional file 1. Database search. [file 11556_2024_338_MOESM1_ESM.docx]

**Supplementary Materials**

**Additional file 1- Database search**

**PubMed**

| **Search** | **Query** |
| --- | --- |
| #1 | Search: breathing exercises[MeSH Terms] |
| #2 | Search: ((((((((((Breathing Exercises[Text Word]) OR (respiratory strength training[Text Word])) OR (inspiratory strength training[Text Word])) OR (expiratory strength training[Text Word])) OR (respiratory muscle training[Text Word])) OR (RMT[Text Word])) OR (inspiratory muscle training[Text Word])) OR (IMT[Text Word])) OR (expiratory muscle training[Text Word])) OR (EMT[Text Word])) OR (breathing muscle training[Text Word]) |
| #3 | #1 OR #2 |
| #4 | Search: stroke [MeSH Terms] |
| #5 | Search: (((((((stroke[Text Word]) OR (acute stroke[Text Word])) OR (sub-acute stroke[Text Word])) OR (early stroke[Text Word])) OR (cerebrovascular accident [Text Word])) OR (stroke[Text Word])) OR (cerebral stroke[Text Word])) OR (CVA[Text Word]) |
| #6 | #4 OR #5 |
| #7 | Search: ((((((((((((((((((respiratory function[Text Word]) OR (respiratory muscle strength[Text Word])) OR (maximum inspiratory pressure[Text Word])) OR (MIP[Text Word])) OR (maximum expiratory pressure[Text Word])) OR (MEP[Text Word])) OR (respiratory muscle endurance[Text Word])) OR (pulmonary function testing[Text Word])) OR (Peak Expiratory Flow[Text Word])) OR (PEF[Text Word])) OR (Forced Expiratory Volume in 1s[Text Word])) OR (FEV1[Text Word])) OR (Forced Vital Capacity[Text Word])) OR (FVC[Text Word])) OR (dyspnea fatigue score[Text Word])) OR (functional capacity[Text Word])) OR (6-minute walking test[Text Word])) OR (Fugl-Meyer Assessment[Text Word])) OR (Functional Ambulation Category[Text Word]) |
| #8 | #3 AND # 6 AND #7 |
| #9 | #8 Filters: Randomized Controlled Trial |

**Embase**

| **Search** | **Query** |
| --- | --- |
| #1 | 'breathing exercise'/exp |
| #2 | 'respiratory strength training':ti,ab,kw OR 'inspiratory strength training':ti,ab,kw OR 'expiratory strength training':ti,ab,kw OR 'respiratory muscle training':ti,ab,kw OR rmt:ti,ab,kw OR 'inspiratory muscle training':ti,ab,kw OR imt:ti,ab,kw OR 'expiratory muscle training':ti,ab,kw OR emt:ti,ab,kw OR 'breathing muscle training':ti,ab,kw OR 'breathing exercises':ti,ab,kw |
| #3 | #1 OR #2 |
| #4 | 'cerebrovascular accident'/exp |
| #5 | 'acute stroke':ti,ab,kw OR 'sub-acute stroke':ti,ab,kw OR 'early stroke':ti,ab,kw OR 'cerebrovascular accident':ti,ab,kw OR stroke:ti,ab,kw OR 'cerebral stroke':ti,ab,kw OR cva:ti,ab,kw |
| #6 | #4 OR #5 |
| #7 | 'respiratory function':ti,ab,kw OR 'respiratory muscle strength':ti,ab,kw OR 'maximum inspiratory pressure':ti,ab,kw OR mip:ti,ab,kw OR 'maximum expiratory pressure':ti,ab,kw OR mep:ti,ab,kw OR 'respiratory muscle endurance':ti,ab,kw OR 'pulmonary function testing':ti,ab,kw OR 'peak expiratory flow':ti,ab,kw OR pef:ti,ab,kw OR 'forced expiratory volume in 1s':ti,ab,kw OR fev1:ti,ab,kw OR 'forced vital capacity':ti,ab,kw OR fvc:ti,ab,kw OR 'dyspnea fatigue score':ti,ab,kw OR 'functional capacity':ti,ab,kw OR '6-minute walking test':ti,ab,kw OR 'fugl-meyer assessment':ti,ab,kw OR 'functional ambulation category':ti,ab,kw |
| #8 | #3 AND # 6 AND #7 |
| #9 | #8 AND 'randomized controlled trial'/de |

**PEDro** (AND, OR and NOT cannot apply as Boolean operators)

| **Search** | **Search Terms** |
| --- | --- |
| S1 | Abstract & Title：Respiratory muscle training Stroke (clinical trial) |
| S2 | Abstract & Title：Inspiratory strength training Stroke (clinical trial) |
| S3 | Abstract & Title：Expiratory strength training Stroke (clinical trial) |
| S4 | Abstract & Title：Respiratory muscle training Stroke (clinical trial) |
| S5 | Abstract & Title：Inspiratory muscle training Stroke (clinical trial) |
| S6 | Abstract & Title：Expiratory muscle training Stroke (clinical trial) |
| S7 | Abstract & Title：Breathing muscle training Stroke (clinical trial) |
| S8 | Abstract & Title：breathing Exercises Stroke (clinical trial) |

**AMED**

| **Search** | **Search Terms** |
| --- | --- |
| S1 | KW respiratory strength training OR KW inspiratory strength training OR KW expiratory strength training OR KW respiratory muscle training OR KW RMT OR KW inspiratory muscle training OR KW IMT OR KW expiratory muscle training OR KW EMT OR KW breathing muscle training OR KW breathing exercises |
| S2 | KW acute stroke OR KW sub-acute stroke OR KW early stroke OR KW cerebrovascular accident OR KW stroke OR KW cerebral stroke OR KW CVA |
| S3 | KW respiratory function OR KW respiratory muscle strength OR KW maximum inspiratory pressure OR KW MIP OR KW maximum expiratory pressure OR KW MEP OR KW respiratory muscle endurance OR KW pulmonary function testing OR KW peak expiratory flow OR KW PEF OR KW forced expiratory volume in 1s OR KW FEV1 OR KW forced vital capacity OR KW FVC OR KW dyspnea fatigue score OR KW functional capacity OR KW 6-minute walking test OR KW Fugl-Meyer assessment OR KW functional ambulation category |
| S4 | S1 AND S2 AND S3 |
| S5 | S4 AND KW random* control* trials |

**CINAHL**

| **Search** | **Search Terms** |
| --- | --- |
| S1 | MH respiratory strength training OR MH inspiratory strength training OR MH expiratory strength training OR MH respiratory muscle training OR MH RMT OR MH inspiratory muscle training OR MH IMT OR MH expiratory muscle training OR MH EMT OR MH breathing muscle training OR MH breathing exercises |
| S2 | MH acute stroke OR MH sub-acute stroke OR MH early stroke OR MH cerebrovascular accident OR MH stroke OR MH cerebral stroke OR MH CVA |
| S3 | MH respiratory function OR MH respiratory muscle strength OR MH maximum inspiratory pressure OR MH MIP OR MH maximum expiratory pressure OR MH MEP OR MH respiratory muscle endurance OR MH pulmonary function testing OR MH peak expiratory flow OR MH PEF OR MH forced expiratory volume in 1s OR MH FEV1 OR MH forced vital capacity OR MH FVC OR MH dyspnea fatigue score OR MH functional capacity OR MH 6-minute walking test OR MH Fugl-Meyer assessment OR MH functional ambulation category |
| S4 | S1 AND S2 AND S3 |
| S5 | S4 AND MH random* control* trials |

**China National Knowledge Infrastructure**

| **Search** | **Search Terms** |
| --- | --- |
| S1 | (Keyword：respiratory strength training) OR (Keyword：inspiratory strength training) OR (Keyword：expiratory strength training) OR (Keyword：respiratory muscle training) OR (Keyword: RMT) OR (Keyword：inspiratory muscle training) OR (Keyword：IMT) OR (Keyword: expiratory muscle training) OR (Keyword：EMT) OR (Keyword: breathing muscle training) OR (Keyword：breathing Exercises) |
| S2 | (Keyword: acute stroke) OR (Keyword：sub-acute stroke) OR (Keyword：early stroke) OR (Keyword：cerebrovascular accident) OR (Keyword: stroke) OR (Keyword：cerebral stroke) OR (Keyword: CVA) |
| S3 | (Keyword: respiratory function) OR (Keyword: respiratory muscle strength) OR (Keyword: maximum inspiratory pressure) OR (Keyword: MIP) OR (Keyword: maximum expiratory pressure) OR (Keyword: MEP) OR (Keyword: respiratory muscle endurance) OR (Keyword: pulmonary function testing) OR (Keyword: peak expiratory flow) OR (Keyword: PEF) OR (Keyword: forced expiratory volume in 1s) OR (Keyword: FEV1) OR (Keyword: forced vital capacity) OR (Keyword: FVC) OR (Keyword: dyspnea fatigue score) OR (Keyword: functional capacity) OR (Keyword: 6-minute walking test) OR (Keyword: Fugl-Meyer Assessment) OR (Keyword: functional ambulation category) |
| S4 | S1 AND S 2 AND S3 |
| S5 | S4 AND (Keyword: random* control* trials) |

**ScienceDirect**

| **Search** | **Search Terms** |
| --- | --- |
| S1 | Title, abstract, keywords: (respiratory strength training OR inspiratory strength training OR expiratory strength training) AND (acute stroke OR stroke) AND (respiratory function OR pulmonary function OR functional capacity) |
| S2 | Title, abstract, keywords:  (respiratory muscle training OR inspiratory muscle training OR expiratory muscle training) AND (acute stroke OR stroke) AND (respiratory function OR pulmonary function OR functional capacity) |
| S3 | Title, abstract, keywords: (breathing muscle training OR breathing exercises) AND (acute stroke OR stroke) AND (respiratory function OR pulmonary function OR functional capacity) |
| S4 | Filters: Research articles |
